# Cough in chronic obstructive pulmonary disease: is it important and what are the effects of treatment?

**DOI:** 10.1186/1745-9974-9-17

**Published:** 2013-06-24

**Authors:** Peter MA Calverley

**Affiliations:** 1 Clinical Sciences Centre, University Hospital Aintree, Lower Lane, L9, 7AL, Liverpool, United Kingdom; 2 University of Liverpool, Lower Lane, L9, 7AL, Liverpool, United Kingdom

**Keywords:** Cough, Chronic Obstructive Pulmonary Disease, Mucus, Bronchitis, Treatment

## Abstract

Over the last 40 years the assessment and treatment of chronic obstructive pulmonary disease has focused primarily on airflow obstruction with little significance given to the problem of cough. The reasons for this include a view that cough arises simply from the direct irritant and inflammatory effect of cigarette smoke or the presence of excess mucus in the airways. Doubt that cough is of any consequence to patients or responsive to current therapies has reinforced this opinion. At odds with this is the emerging evidence that cough impacts adversely on patients’ health status and forms an important component of recently validated quality of life instruments. This article presents the arguments why the assessment and treatment of cough should have a more prominent place in the clinical management of COPD.

## Introduction

Chronic obstructive pulmonary disease (COPD) is now recognised to be a condition of global importance in terms of its impact on the morbidity and risk of premature death of millions of people. The definition of COPD continues to be refined [1]. However there is general agreement that this condition results from the interplay of several respiratory pathologies with emphysema and respiratory bronchiolitis being the most important which lead to persistent and usually progressive airflow obstruction. This formulation is rather different from the way in which the same clinical problem was viewed only 40 years ago when a symptomatic definition of chronic cough and sputum production was the hallmark of an illness which progressed to disabling breathlessness and ultimately death from right heat failure (‘blue and bloated’ bronchitic COPD). The reasons for this change of emphasis are too complex to explain in detail here but they undoubtedly led to the clinical significance of cough as a symptom in COPD being under-appreciated.

The role of cough in COPD has been well reviewed before [2]. That review was conducted from the perspective of an expert on cough research but this brief review takes a slightly different approach seeing cough as one part of the problems experienced by the COPD patient. This article will examine whether the currently accepted view that cough is of little significance in COPD is correct by considering the arguments put forward by those sceptical of the relevance of cough to COPD. The outcome of such an approach may surprise some unfamiliar with the growing body of literature relevant to this field.

### ‘It’s a smoker’s cough of no great significance’

We do not have good data about the frequency with which smokers cough. This reflects one of the major problems in cough research – the lack of simple metrics which describe coughing. How often should the subject cough and with what intensity before it is classified as significant? This Gordian knot was cut by the Medical Research Council in the 1960’s when they developed an epidemiological definition of chronic bronchitis [3]. They had previously graded cough as productive or unproductive and for their definition they focussed on cough present for at least 3 months of 2 consecutive years, thereby emphasising chronicity and reasonably enough believing that chronic mucus production marked out a clinically more severe event. The recognition that deaths from chronic bronchitis were much more frequent in smokers [4] emphasised the link between bronchitis, smoking and adverse outcomes while compelling data that smoking cessation decreased both bronchitic symptoms and mortality suggested that the cough was merely an epiphenomenon and not causally relevant.

This analysis was accepted for much of the last 3 decades but there are now reasons to doubt it. Coughing is associated with airway inflammation in non-smoking asthmatics and there are objective data showing persistence of airway inflammation in ex-smoking COPD patients [5], likely reflecting different mechanisms of inflammation at different stages in the evolution of the pulmonary pathology [6]. Many clinical trials have identified large numbers so patients with bronchitic symptoms as part of their physiologically defined COPD and in some studies patients have been selected on the basis of chronic cough being present [7]. While cough diminishes and even disappears in many ex-smokers this is not the case in many of those where more severe airflow obstruction has developed.

### Cough in COPD is just due to the accumulation of mucus

**Figure 1:**
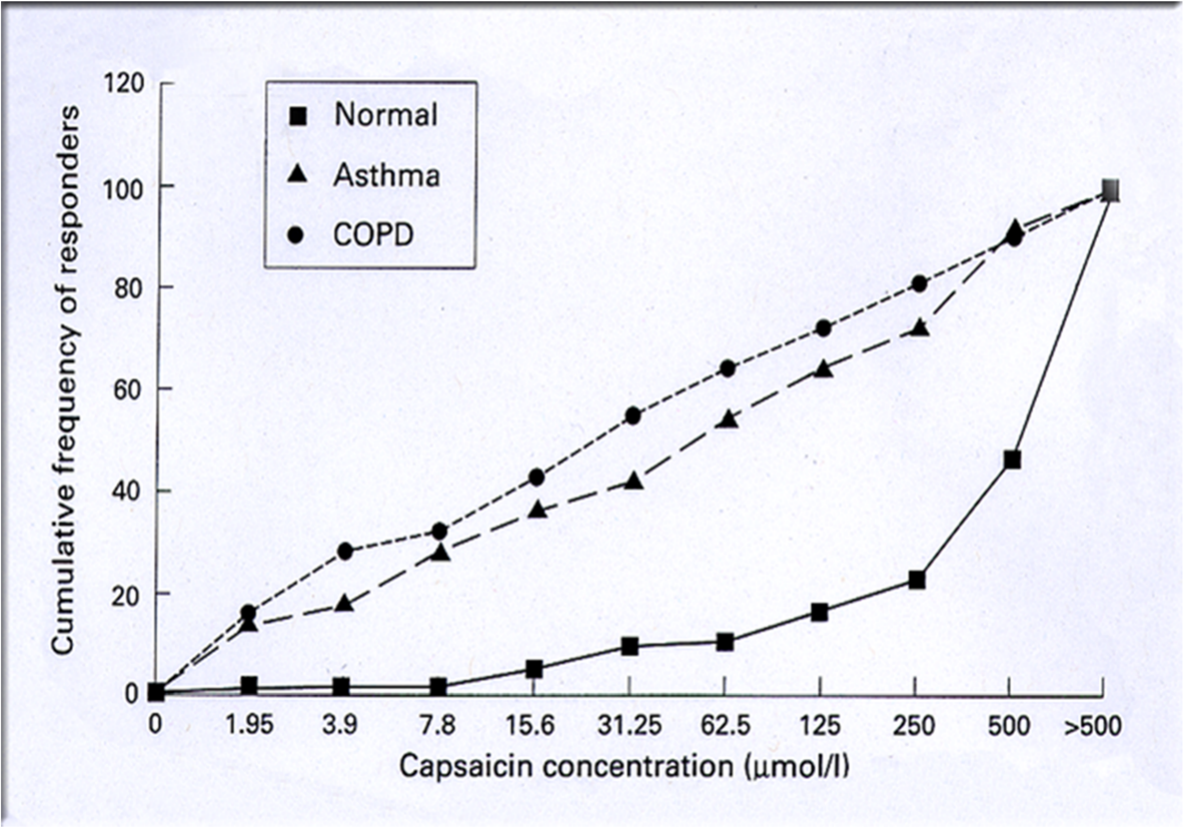
**Capsaicin cough sensitivity in COPD, asthma and healthy subjects.** The cumulative frequency of responders defined as 5 coughs evoked by that concentration of inhaled capsaicin. Populations reflect healthy normal subjects, and patients with COPD or chronic stable asthma.

**Figure 2:**
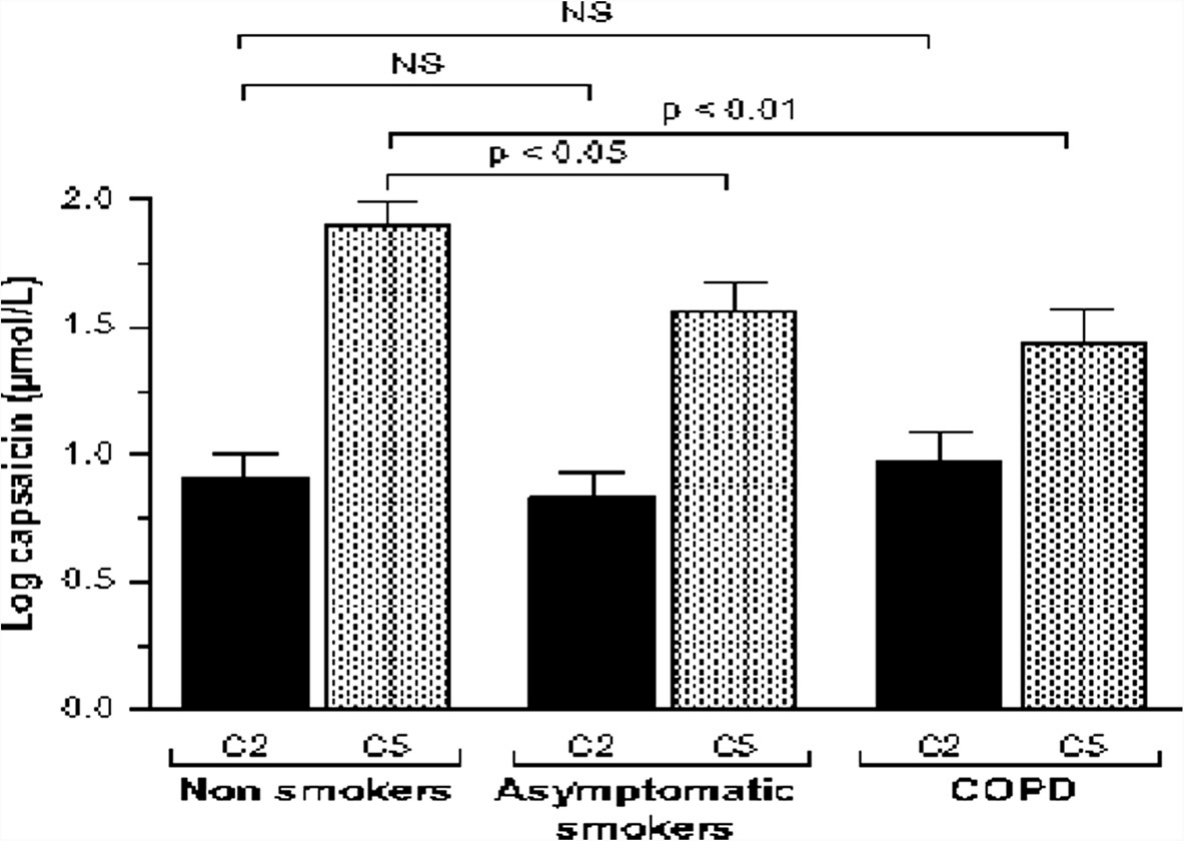
**Capsaicin cough sensitivity in COPD patients without cough, asymptomatic cougher and healthy subjects.** Mean log capsaicin concentrations in 92 healthy subjects, 68 asymptomatic smokers and 42 COPD patients not complaining of cough. Note that the concentration required to induce 5 coughs is lower in the COPD patients even though they did not report troublesome cough. The values are similar to those in the smoking controls and lower than in the healthy subjects.

The epidemiological definition of chronic bronchitis emphasised the association with sputum production and it seemed reasonable to assume that excess mucus accumulation was the main factor driving cough in COPD. This agreed with pathological observation by Reid and Heard of mucous gland hypertrophy in the central airways of patients dying with what we now term COPD [8]. Loss of ciliary structure and associated impairments of muco-ciliary clearance were described in bronchitic patients as was a favourable effect of beta-agonists in improving this defect [9]. However other mechanisms also play a role and there is now evidence for enhanced non-specific neutrally mediated cough responsiveness in COPD patients similar to that seen in adult asthmatics (Figure 1) [10]. These studies used the C5 response to inhaled capsaicin as their marker of responsiveness rather than the C2 response where the difference between patients and control subjects was less evident. These data have recently been confirmed in a larger study of healthy subjects, asymptomatic smokers and patients with moderately severe COPD (Figure 2), [11]. As in the Liverpool data the C5 rather than the C2 distinguished between the groups, although here the smokers and COPD patients showed similarly heightened cough responses. This suggests that cough reflex sensitivity is enhanced in COPD but not to the same degree as is seen in idiopathic cough and that mucus production which was not a characteristic of these patients is not always needed for an abnormal cough response to be present.

### Self-reported cough is not important to COPD patients

The realisation of the association between self-reported cough and deaths from bronchitis led to the ‘British hypothesis’ that disease progression in COPD was related to the presence of bronchitic symptoms. This was tested in the long term study of lung function, symptoms and mortality in British postal workers which failed to find any association with disease progression and bronchitis [12]. Coupled with the earlier negative studies of chronic tetracycline treatment in reducing clinical deterioration in people with more advanced disease, there was a general feeling that cough was of little consequence in COPD. In the manner of the times this decision was made without asking the patients what they thought.

While there is no doubt that breathlessness is the most dramatic and disabling symptom experienced by COPD patients, more recent data shows that cough is also a matter of concern. Danish data in a large population study found that the presence of chronic mucous hypersecretion increased the risk of having pneumonia and was associated with a faster decline in lung function in men [13]. More recently workers in Switzerland found that people with very mild airflow obstruction who had symptoms, principally cough and sputum production, showed accelerated decline in lung function over time, a finding not seen in patients with mild airflow obstruction who had no symptoms [14].

The impact of cough on patient well being has now been evaluated. In a telephone survey of 2950 COPD patients Kessler et al. found that cough was reported by 55% of subjects with 20% rating it severe to extreme [15]. These symptoms were most evident on rising in the morning and were more likely to be present then than at other times of the day. Finally specific questions about the impact of cough on daily life form a part of the St Georges Respiratory questionnaire, one of the best validated health status measurements in the COPD field [16]. Cough is also one of the 8 questions identified as providing important information independent of the other domains in the COPD Assessment Test (CAT) another psychometrically validated instrument to assess disease severity in COPD [17]. Clearly patients find cough to be an important symptom even if their doctors are yet to be convinced.

### A history of cough provides no clinically useful information

Cough is now considered, along with breathlessness and sputum production, to be one of the cardinal symptoms of COPD [1] and which taken together with exposure to a suitable risk factor should lead to a diagnostic spirometry test. The presence of cough increases the chances that a screening spirometry in primary care will be positive [18]. As noted above a question about cough (I never cough – I cough all the time) contributes independently to the CAT score which is now being widely used to assess the impact of COPD on the patient’s well being. Exacerbations are key events in the natural history of COPD and patients who exacerbate frequently form a discrete phenotype of patient [19]. Several groups have found that the presence of cough and sputum identifies patients at greater risk of subsequent exacerbation as defined by the need for antibiotics and/or corticosteroids [20]. Questions about cough form an important part of the recently validated EXACT-PRO questionnaire used to monitor patients for the onset and resolution of these events [21].

### Cough does not respond to treatment

At present it is difficult to quantify the degree to which cough responds to treatment for COPD. In part this reflects the lack of focus on cough in clinical studies where most attention has been paid to the relief of breathlessness or the prevention of exacerbations. However the lack of a validated measure of cough intensity and frequency which is known to respond to interventions is also a factor. This situation may improve when newer instruments such as the CAT and EXACT questionnaires are studied in treatment trials.

There is general agreement that COPD patients who stop smoking are less likely to report the symptom of cough although this change may be more evident in the earlier stages of the disease, when lung function improvement with treatment is more evident. The proportion of patients with mild-moderate COPD who report a chronic cough declines by about 5% over a 5 year follow up period but only 10% of similar patients who quit complain of cough 5 years after stopping [22]. Whether other therapies such as pulmonary rehabilitation influence cough frequency is not known. Treatment with non-specific cough suppressants has proven to be disappointing. In a carefully designed cross over study 21 COPD patients received 60 mg oral codeine twice daily or an identical placebo and assessed at baseline and on two study days each one week apart. Subjectively reported cough and objective counts of the number of coughs and their duration were made together with a measurement of citric acid induced cough threshold [23]. Although most patients reported spontaneous improvement in their symptoms there were no differences in the subjective or objective treatment arms of the study. Uncontrolled observations of capsaicin cough threshold between patients taking regular Ipratropium and those not treated with this anticholinergic agent suggested that the cough threshold was lower in the treated patients [10]. However this may reflect confounding by treatment indication rather than a true drug effect.

Many pharmaceutical studies of COPD treatment have recorded cough as a symptom in daily patient diary cards but have seldom analysed the resulting data. One example of this is presented in Table 1 which is derived from the TRISTAN study comparing an inhaled corticosteroids, a long-acting beta-agonist, the combination SFC and placebo in severe COPD patients over one year [24]. The diary card reported cough on a simple intensity scale and these results make no adjustment for the number of people in whom the symptom is not reported. Nonetheless it is clear that patients receiving treatment reported less cough although the difference was only statistically significant when both inhaled corticosteroid and beta-agonist were co-administered. Given the focus of new anti-inflammatory therapies like roflumilast on patients who report cough [25] it is to be hoped that future studies using these agents will determine whether cough improves with therapy.

**Table 1:** Adjusted mean scores from daily diary card recordings of cough (0–3 intensity scale) from the TRISTAN study (24)

	**Adjusted mean**	**Treatment effect (SFC-comparator)**	**95% CL**	**p-value**
Placebo				
(n = 357)	1.44	−0.091	−0.17, -0.02	0.018
Salmeterol				
(n = 370)	1.36	−0.018	−0.09, 0.06	0.639
FP				
(n = 368)	1.38	−0.037	−0.11, 0.04	0.340
SFC				
(n = 351)	1.35	-	-	-

Note that not all patients report cough and the scale not being validated for its measurement properties. Despite this the combination of bronchodilator and inhaled corticosteroid was associated with significantly less cough during the one year study period.

*FP* Fluticasone propionate.

*SFC* Salmeterol Fluticasone propionate Combination.

In summary it appears that the presence of cough with or without sputum is an early feature of clinical COPD that helps identify people at risk of progressive disease. The cough reflex in COPD is increased to levels seen in current smokers even when the patient no longer smokes. Cough remains a key symptom in the diagnosis of COPD and its presence helps identify those patients at greatest risk of future exacerbations. Smoking cessation early in the natural history of the disease remains the most effective way of reducing cough but other therapies show some promise. Non-specific cough suppressants do not appear effective in COPD and should be avoided. What is really needed is a more systematic study of this important symptom in larger numbers of well characterised patients using validated ways of assessing the occurrence and impact of cough. Once we better understand the nature and variability of this symptom at a patient level then we will be able to develop more effective methods of managing it in clinical practice.

## Abbreviations

COPD: Chronic Obstructive Pulmonary Disease; C2: Concentration of capsaicin inducing 2 coughs; C5: Concentration of capsaicin inducing 5 coughs; CAT: COPD Assessment Test; EXACT-PRO: EXAcerbations of Chronic Obstructive Pulmonary Disease Tool (EXACT): a patient-reported outcome (PRO) measure; TRISTAN: Trial of Inhaled Steroids and long-acting β2 agonists Competing Interests.

## Competing interests

The author declares that they have no competing interests.
